# Relationship between the sense of nursing professional pride and adversity quotient, grit levels among nurses in blood purification centers: a multicenter cross-sectional study

**DOI:** 10.3389/fpsyg.2024.1441010

**Published:** 2024-11-12

**Authors:** Wenbin Xu, Lin Li, Qian Jiang, Yiqian Fang, Qian Yang

**Affiliations:** School of Nursing, Chengdu Medical College, Chengdu, Sichuan, China

**Keywords:** blood purification center nurses, professional pride, adversity quotient, grit, correlation analysis

## Abstract

**Objective:**

This study aims to examine nursing professional pride levels in blood purification center nurses, explore its relationship with adversity quotient and grit, and identify influencing factors.

**Methods:**

Using convenience sampling, this study selected 231 nurses from blood purification centers at 11 tertiary hospitals in China during July to August 2023 as research subjects. A general data survey questionnaire, nurse professional pride scale, nurse adversity quotient self-assessment scale, and the Oviedo resilience scale were employed for the investigation. The results were statistically analyzed using descriptive analysis, *t*-tests, one-way ANOVA, Pearson correlation analysis, and multiple stepwise linear regression analysis.

**Results:**

In the blood purification center, the average scores were as follows: nursing professional pride (69.53 ± 15.21), adversity quotient (132.90 ± 41.10), and grit (30.95 ± 10.54). There was a significant positive correlation between nursing professional pride, adversity quotient, and grit (*P* < 0.001). Multiple linear regression analysis (*n* = 231) revealed that education background, professional title, average monthly income, adversity quotient, and grit significantly influenced nursing professional pride (*P* < 0.001). The *R*^2^ value indicates that this study accounted for 76.2% of the total variance in nursing professional pride.

**Conclusion:**

The findings of this study indicate that blood purification center nurses have a moderate level of nursing professional pride. The level of nursing professional pride can be enhanced by focusing on factors such as adversity quotient and grit.

**Registration:**

This study was approved by the Ethics Committee of Pidu District People's Hospital, Chengdu, China (2021 No. 231). All participants were informed of the relevant information and research purposes before their participation. Participants were also required to independently and anonymously complete the questionnaires.

## 1 Introduction

Nurses, as healthcare professionals within the healthcare system, provide substantial care services with the objective of enhancing human health. However, due to long working hours, high job stress, irregular schedules, and inadequate compensation, nurses often exhibit low professional pride, insufficient job satisfaction, and consequently experience professional burnout and a tendency to leave their profession (Vermeir et al., 2018; Msiska et al., 2014; Meng et al., 2015). The blood purification center is a highly specialized and enclosed environment that requires nurses to work in a high-intensity mode for extended periods each day. Additionally, hemodialysis patients rely on dialysis treatment year-round for their survival, necessitating that hemodialysis nurses ensure the quality and safety of patient dialysis treatment while also focusing on patients' self-health management during non-dialysis hours. Moreover, they must assist physicians in managing various emergencies and preventing complications during the hemodialysis process (Ghanayem et al., 2020; Cao and Chen, 2021). A multitude of factors contribute to an elevated workload, which, in extreme cases, can have a detrimental impact on the psychological wellbeing of hemodialysis nurses. This undoubtedly exacerbates the stress burden on hemodialysis nurses, which in turn leads to a decrease in their professional pride and ultimately results in a decline in the quality of care. Consequently, it is of paramount importance to ascertain the current levels of nursing professional pride among nurses in blood purification centers, identify the influencing factors, enhance nursing professional pride, improve nursing quality, and ensure the safety of dialysis patients.

Nursing professional pride is defined as the positive emotional state experienced by nurses after successfully performing their duties. Professional pride serves to strengthen and elevate the nurses' professional status, enhance their sense of professional identity, and increase job satisfaction (Jeon et al., 2020). Empirical studies have demonstrated that individuals with high levels of professional pride exhibit higher levels of happiness (Sneltvedt and Sørlie, 2012). They are highly satisfied with their work and exhibit trust and a positive attitude toward their profession (Honingh et al., 2012). They are inclined to take proactive actions and exert efforts to achieve success (Herrald and Tomaka, 2002). By adopting a holistic approach to assessing the needs of both patients and their families, nurses provide comprehensive care that safeguards and maintains their health. This contributes to nurses' positive perception of professional pride (Vikström and Johansson, 2019; Kim and Sim, 2020). When nurses experience higher levels of professional pride and efficacy in their work, they tend to invest their full enthusiasm and positive emotions into their duties, thereby delivering high-quality nursing services (Ren et al., 2021). Zhan et al. (2020) discovered through surveys of nursing students with different educational backgrounds that married students with high stress levels and low family support exhibited lower levels of professional pride and a lower willingness to engage in frontline work. Tjoflåt et al. (2018) conducted qualitative interviews with nurses in Tanzanian hospitals and discovered that they exhibited a profound sense of professional pride and responsibility. In their study of older adult care nurses, Vikström and Johansson (2019) found that these nurses exhibited high levels of professional pride. This was attributed to their ability to provide care for the older adult through their own skills, which gave them a sense of accomplishment. Qin et al. (2018) found that pediatric nurses exhibited a high level of professional pride, with a moderate level of professional identity. In conclusion, the current level of nursing professional pride exhibits considerable variability, and further research is necessary to provide a foundation for the development of intervention measures designed to enhance the overall level of nursing professional pride.

However, despite the aspects of nursing work that are pride-inducing, it can still be influenced by societal, environmental, and individual factors. For example, factors such as an environment that prioritizes hierarchy over physical and mental wellbeing, tasks outside of the core responsibilities, low wages, and other adverse personal experiences (Valizadeh et al., 2018; Obeidat et al., 2018; Vander Elst et al., 2016). Bildt and Michélsen (2002) found that poor mental health is associated with low professional pride. Psychological issues such as a sense of worthlessness, depression, professional burnout, low job satisfaction, and lack of motivation can diminish nursing professional pride, resulting in reduced work efficiency, increased absenteeism, and a desire to resign (Green et al., 2020; Höld et al., 2020; Nishimoto et al., 2022). Professional burnout and job dissatisfaction can have a negative impact on nurses' psychological wellbeing and the quality of care, which may result in adverse health outcomes for patients (Modaresnezhad et al., 2021; Shi et al., 2021; Wood et al., 2021). Additionally, low nursing quality can affect various aspects, including individuals, families, and society, leading to increased employee turnover, healthcare costs, and workload. This may result in nurses being perceived as incompetent (Mudallal et al., 2017). Furthermore, excessive stress can erode nursing professional pride, diminish the quality of professional life, and increase turnover rates (Chegini et al., 2019). Consequently, it is of paramount importance to ensure that nurses take pride in their profession and have sufficient satisfaction and motivation in order to improve the quality of nursing care. However, there is currently a paucity of research that is specifically focused on nursing professional pride, and the factors that influence it are not yet fully understood. Consequently, this study endeavors to elucidate the factors that influence nursing professional pride, thereby expanding the domain of research on professional pride. The broaden-and-build theory of positive emotions suggests that positive emotions can expand an individual's immediate thought-behavior patterns, helping them build sufficient and sustainable physiological and psychological resources to enhance their sense of pleasure and purpose (Fredrickson, 1998). As a form of positive emotion, professional pride not only helps individuals enhance self-esteem and motivation but also encourages engagement in prosocial behaviors. Currently, research on nurses' professional pride is limited and lacks a comprehensive exploration of its influencing factors. Therefore, this study aims to delve into the influencing factors of nurses' professional pride, expanding the research scope in this area.

Empirical evidence suggests that adversity quotient may act as a protective factor against the intention to resign, enhance job satisfaction, and strengthen job identification (Aryani et al., 2021). Furthermore, adversity quotient is inversely correlated with professional stress and feelings of professional burnout (Yu, 2021; Ikbar et al., 2022). Consequently, elevated levels of professional stress and burnout can result in a decline in nursing professional pride. In addition, it has been demonstrated that the quality of grit can reduce professional burnout, enhance job satisfaction, and improve the quality of nursing care (Shin, 2022). Therefore, it is worth exploring the correlation between adversity quotient, grit, and nursing professional pride, as well as investigating the influencing factors of nursing professional pride. This can provide a more comprehensive theoretical basis for developing intervention measures to enhance the level of nursing professional pride.

The adversity quotient, proposed by American vocational training specialist Paul Stoltz in the 1990s, refers to an individual's ability to face setbacks and adversity with courage, willingness to overcome adversity, and ability to overcome difficulties (Mohd Adnan and Mohd Matore, 2022). It is an important indicator of an individual's psychological resilience. People with high adversity quotient can face difficulties and challenges with a positive and optimistic attitude and ultimately triumph over adversity (Ikbar et al., 2022). Currently, many nurses face various adversities such as confusion about career development, lack of energy, and mental health problems due to constraints related to public bias, workload, and hospital management. Research indicates that the level of adversity quotient among nurses not only affects their physical and mental wellbeing, but also has an impact on the stability of the nursing workforce (Zhao and Li, 2019). The adversity quotient theory states that individuals respond differently when faced with adversity or setbacks, and adversity quotient can help nurses better fulfill their responsibilities, meet their physical and mental health development needs, enhance their ability to withstand setbacks (Yang et al., 2018), increase nursing professional identity and pride, and ultimately stabilize the nursing management team.

Grit is a positive psychological trait that falls within the realm of positive psychology (Duckworth et al., 2007). Grit can be defined as an individual's persistent pursuit and sustained enthusiasm for a specific goal. On the one hand, grit signifies a person's determination to persevere in the face of setbacks. Conversely, it also signifies an individual's inclination toward having a positive interest in enduring objectives (Eskreis-Winkler et al., 2014). It has been demonstrated that grit plays a pivotal role in healthcare and serves as a significant predictive indicator for achievement and success (Aguirre-Urreta and Hu, 2019). The theory of positive psychology posits that grit is a positive personality trait that plays a pivotal role in the psychological wellbeing, job performance, job satisfaction, subjective wellbeing, and overall performance of nurses (Seguin, 2019; Cho and Kim, 2022; Khorram-Manesh and Goniewicz, 2023; Halperin and Eldar Regev, 2021; Sellers, 2019; Jeong et al., 2019). This may contribute to an increase in nursing professional pride, a reduction in employee turnover rates, and an improvement in the quality of nursing care.

In conclusion, the impact of adversity quotient and grit on the level of nursing professional pride among hemodialysis nurses is currently under-researched, both domestically and internationally. Consequently, this study focuses on nurses employed in hemodialysis centers and investigates their levels of nursing professional pride. Additionally, it analyzes the correlation between nursing professional pride and adversity quotient as well as grit. The objective is to provide nursing managers with insights from the perspective of adversity quotient and grit, enabling them to develop intervention measures to enhance nursing professional pride, improve job satisfaction, enhance the quality of clinical nursing care, and reduce feelings of professional burnout.

## 2 Article types

This is a cross-sectional study to explore the relationship among professional pride, adversity quotient and perseverance level, and to determine the influencing factors of professional pride of nurses in blood purification center.

## 3 Materials and methods

### 3.1 Study design and participants

In this cross-sectional survey conducted from July to August 2023, a convenience sampling method was used to select nurses from the blood purification center of 11 tertiary A-grade hospitals in China as research subjects. The inclusion criteria were as follows: (1) Registered clinical nurses currently employed; (2) Nurses with at least one year of work experience in the blood purification center; (3) Volunteers willing to participate. The exclusion criteria were as follows: (1) Nursing professionals from other hospitals visiting for observational or educational purposes; (2) Nurses on maternity leave, personal leave, or engaged in off-site learning and not currently on duty. This study has been approved by the Ethics Committee of Pidu District People's Hospital (Approval No: 2021, 231). All participants were informed about the relevant information and purpose of the research before participating. They were also required to complete the questionnaire independently and anonymously.

### 3.2 Data collection

After obtaining approval from the nursing supervisors of each hospital's blood purification center, we assigned 1–2 investigators to each hospital. After informing the participants of the survey's purpose and the estimated completion time (15–25 min) and obtaining their informed consent, we conducted the survey using a paper-based questionnaire. A total of 250 questionnaires were distributed in this survey, and 231 valid questionnaires were collected, resulting in a response rate of 92.4%.

### 3.3 Measurements

#### 3.3.1 General information survey questionnaire

The questionnaire was designed by the research team after reviewing a significant amount of literature and discussions. It consisted of demographic information, including age, sex, presence of children, education background, only child status, marital status, length of professional service, professional title, employment type, average monthly income, and whether they had received training related to psychology, totaling 11 items.

#### 3.3.2 Chinese version of nursing professional pride scale

This scale was developed by Korean scholars Jeon et al. (2020), and later translated into Chinese by Chinese scholars including Jeong et al. (2019). It consists of 22 items categorized into 5 dimensions: role satisfaction (6 items); professional sense (6 items); job retention intention (5 items); problem solver role (6 items); and self-achievement (4 items). The scale employs a 4-point Likert scoring system, where 1 indicates “completely disagree” and 4 indicates “completely agree.” In this study, a positive scoring method was used, with higher scores indicating a higher level of professional pride among nurses. The scale demonstrated good internal consistency, with a Cronbach's α coefficient of 0.923.

#### 3.3.3 The self-evaluation scale for nurses' adversity quotient

This scale was developed by scholars including Liu et al. (2022), using Paul Stoltz's concept of adversity quotient (Stoltz and Wiley, 1997) as a framework. It consists of 44 items categorized into 4 dimensions: adversity control (12 items); adversity attribution (8 items); adversity impact (14 items); and adversity endurance (10 items). The scale employs a 5-point Likert scoring system, with 1 indicating “completely inconsistent” and 5 indicating “completely consistent.” Higher scores indicate a lower level of adversity quotient. However, in this study, a reversed scoring method was used. In the overall data of this study, the results of each item on the scale were reversed to positive scoring, so higher scores indicate a higher level of adversity quotient. The scale exhibited good reliability and validity, with a Cronbach's α coefficient of 0.897.

#### 3.3.4 The oviedo grit scale

This scale was developed by scholars including Postigo et al. (2021), combining classical measurement theory and item response theory. It was later translated into Chinese by Chinese scholars including Liu et al. (2023). It consists of 10 items and represents a single dimension. The scale employs a 5-point Likert scoring system, with 1 indicating “completely disagree” and 5 indicating “completely agree.” In this study, a positive scoring method was used, with higher scores indicating a higher level of perseverance. The scale showed good reliability and validity, with a Cronbach's α coefficient of 0.937.

### 3.4 Data analyses

This study conducted data analysis using IBM SPSS 26.0 (IBM Corp., Armonk, NY, USA). Initially, descriptive analysis was performed on all data (utilizing percentages, means, and standard deviations). Independent sample *t*-tests and one-way analysis of variance (ANOVA) were employed to compare the differences in nursing professional pride based on various demographic factors. Subsequently, Pearson correlation analysis was used to examine the relationships between nursing professional pride, adversity quotient, and grit. Finally, multiple stepwise linear regression analysis was employed to identify the factors influencing nursing professional pride.

## 4 Results

### 4.1 Demographic characteristics and analysis of factors related to nursing professional pride

The demographic characteristics of the participants and features related to nursing professional pride are detailed in Table 1. Among the 231 nurses who participated in this study, a significant majority were female (79.2%), most of them had children (74.9%), and over half of the participants were married (68.4%). Regarding education, 47.2% held a college degree, while 48.9% held the title of the primary nurse practitioner. Approximately 48.9% reported a monthly income in the range of 3,000–5,000 yuan. More than half of the participants had not received training in psychology (50.2%).

**Table 1:** Comparison of nursing professional pride among nurses with different socio-demographic characteristics (*n* = 231).

**Socio-demographic characteristics**	***N* (%)**	**Nursing professional pride *M (SD)***	***t/F***	***P***
Sex			0.942	0.470
Male	48 (20.8)	69.88 (14.50)		
Female	183 (79.2)	69.44 (15.42)		
Age (years)			6.507	0.002
18–25	85 (36.8)	65.47 (14.89)		
26–45	105 (45.5)	73.22 (14.19)		
>45	41 (17.7)	68.51 (16.38)		
Marital status			0.361	0.718
Married	158 (68.4)	69.78 (14.58)		
Unmarried	73 (31.6)	69.00 (16.57)		
Presence of children			−1.099	0.273
Yes	173 (74.9)	68.90 (14.89)		
No	58 (25.1)	71.43 (16.10)		
Length of professional service (years)			3.4790	0.009
< 3	32 (13.9)	63.66 (15.01)		
3–5	53 (22.9)	66.57 (14.86)		
6–10	50 (21.6)	73.90 (15.07)		
11–20	55 (23.8)	72.60 (13.45)		
>20	41 (17.7)	68.51 (16.38)		
Education background			48.453	< 0.001
Technical secondary school degree	26 (11.3)	55.31 (16.87)		
Associate degree	109 (47.2)	63.50 (10.01)		
Bachelor degree	82 (35.5)	79.23 (10.14)		
Master degree and higher	14 (6.1)	86.07 (21.75)		
Employment type			−1.966	0.051
Permanent Employment	64 (27.7)	66.38 (15.07)		
Non-permanent Employment	167 (72.3)	70.74 (15.13)		
Professional title			41.639	< 0.001
Nurse	24 (10.4)	54.25 (17.99)		
The primary nurse practitioner	113 (48.9)	64.27 (11.19)		
Nurse-in-charge	80 (34.6)	80.54 (10.13)		
Deputy director nurse or above	14 (6.1)	75.29 (19.01)		
Only child status			1.630	0.105
Yes	126 (54.5)	71.02 (15.55)		
No	105 (45.5)	67.75 (14.52)		
Average monthly income (RMB)			48.288	< 0.001
< 3,000	22 (9.5)	55.18 (18.70)		
3,000–5,000	113 (48.9)	63.19 (11.14)		
5,001–8,000	80 (34.6)	80.53 (8.02)		
>8,000	16 (6.9)	79.06 (21.10)		
Whether they had received training related to psychology			−209	0.834
Yes	115 (49.8)	69.32 (15.87)		
No	116 (50.2)	69.74 (14.59)		

The results of *t*-tests and analysis of variance (ANOVA) showed differences in nursing professional pride concerning age, length of professional service, education background, professional title, and average monthly income (*P* < 0.05).

### 4.2 Scores of nursing professional pride, adversity quotient, and grit among hemodialysiscenter nurses

The P-P plots indicate that nursing professional pride, adversity quotient, and grit approximately follow a normal distribution. Therefore, these variables can be described using means and standard deviations. Details are presented in Table 2. The total average score for nursing professional pride was 69.53 ± 15.21. The average scores for the dimensions of role satisfaction, professional sense, job retention intention, problem solver role, and self-achievement were 15.46 ± 4.03, 15.23 ± 3.96, 13.03 ± 3.34, 15.58 ± 3.84, and 10.23 ± 2.75, respectively. This indicates that the overall nursing professional pride among hemodialysis center nurses is at a moderate level.

**Table 2:** Descriptive analyses of nursing professional pride, adversity quotient and grit (*n* = 231).

**Variables**	***Min*.**	***Max*.**	***M***	***SD***
**Nursing professional pride**				
Role satisfaction	6.00	24.00	15.46	4.03
Professional sense	6.00	24.00	15.23	3.96
Job retention intention	5.00	20.00	13.03	3.34
Problem solver role	6.00	24.00	15.58	3.84
Self-achievement	4.00	16.00	10.23	2.75
Total score	28.00	107.00	69.53	15.21
**Adversity quotient**				
Adversity control	12.00	60.00	35.93	12.00
Adversity attribution	8.00	40.00	23.58	8.62
Adversity impact	14.00	70.00	43.06	14.05
Adversity endurance	10.00	50.00	30.34	10.22
Total score	51.00	214.00	132.90	41.10
**Grit**				
Total score	10.00	50.00	30.95	10.54

The average score for adversity quotient was 132.90 ± 41.10, with scores for the dimensions of adversity control, adversity attribution, adversity impact, and adversity endurance being 35.93 ± 12.00, 23.58 ± 8.62, 43.06 ± 14.05, and 30.34 ± 10.22, respectively. This suggests that the overall adversity quotient among hemodialysis center nurses is at a moderate level.

The average score for grit was 30.95 ± 10.54, indicating that hemodialysis center nurses have a moderate level of resilience.

### 4.3 Analysis of the correlation between nursing professional pride, adversity quotient, and grit among nurses in hemodialysis centers

The results of the correlation analysis showed that there was a significant positive correlation (*P* < 0.001) between nursing professional pride and its four dimensions, adversity quotient and its five dimensions, as well as grit. Specific details can be found in Table 3. Most of the correlation coefficients were between 0.3 and 0.4, indicating a moderate correlation between nursing professional pride, adversity quotient, and grit among nurses in hemodialysis centers.

**Table 3:** Correlation analyses (R-values) between nursing professional pride, adversity quotient and grit (*n* = 231).

**Variables**	**Total score for adversity quotient**	**Adversity control**	**Adversity attribution**	**Adversity impact**	**Adversity endurance**	**Total score for nursing professional pride**	**Role satisfaction**	**Professional sense**	**Job retention intention**	**Problem solver role**	**Self-achievement**	**Total score for grit**
Total score for adversity quotient	1											
Adversity control	0.932^**^	1										
Adversity attribution	0.877^**^	0.767^**^	1									
Adversity impact	0.926^**^	0.813^**^	0.732^**^	1								
Adversity endurance	0.914^**^	0.810^**^	0.778^**^	0.778^**^	1							
Total score for nursing professional pride	0.552^**^	0.516^**^	0.464^**^	0.507^**^	0.528^**^	1						
Role satisfaction	0.443^**^	0.416^**^	0.360^**^	0.402^**^	0.438^**^	0.893^**^	1					
Professional sense	0.493^**^	0.465^**^	0.432^**^	0.444^**^	0.464^**^	0.838^**^	0.638^**^	1				
Job retention intention	0.446^**^	0.396^**^	0.385^**^	0.420^**^	0.426^**^	0.849^**^	0.737^**^	0.636^**^	1			
Problem solver role	0.517^**^	0.515^**^	0.417^**^	0.461^**^	0.490^**^	0.851^**^	0.713^**^	0.637^**^	0.625^**^	1		
Self-achievement	0.431^**^	0.373^**^	0.367^**^	0.420^**^	0.408^**^	0.796^**^	0.663^**^	0.597^**^	0.612^**^	0.592^**^	1	
Total score for grit	0.413^**^	0.380^**^	0.353^**^	0.391^**^	0.380^**^	0.688^**^	0.620^**^	0.558^**^	0.641^**^	0.575^**^	0.510^**^	1

^**^P < 0.001.

### 4.4 Factors influencing nursing professional pride in hemodialysis centers

Using the nursing professional pride score of nurses in hemodialysis centers as the dependent variable, multiple stepwise linear regression analysis was conducted with seven statistically significant variables from the single-factor analysis and correlation analysis as independent variables. The assignment of values is shown in Table 4. The results indicated that education background, professional title, average monthly income, adversity quotient, and grit are factors influencing nursing professional pride. *R*^2^ indicates that this study collectively explained 76.2% of the total variance in nursing professional pride. Details are presented in Table 5.

**Table 4:** Variable assignment status.

**Variables**	**Assignment method**
Age(years)	1 = 18–25, 2 = 26–45, 3 ≥ 45
Length of professional service (years)	1 ≤ 3, 2 = 3–5, 3 = 6–10, 4 = 11–20, 5≥20
Education background	1 = Technical secondary school degree, 2 = Associate degree, 3 = Bachelor degree, 4 = Master degree and higher
Professional title	1 = Nurse, 2 = The primary nurse practitioner, 3 = Nurse-in-charge, 4 = Deputy director nurse or above
Average monthly income (RMB)	1 ≤ 3,000, 2 = 3,000–5,000, 3 = 5,001–8,000, 4≥8,000
Total score for nursing professional pride	Raw value
Total score for adversity quotient	Raw value
Total score for grit	Raw value

**Table 5:** Multivariate linear stepwise regression analysis of nursing professional pride (*n* = 231).

**Variables**	***B***	***SE***	**β**	***t***	***P***
Education background	5.982	0.722	0.3	8.287	< 0.001
Professional title	3.269	0.744	0.161	4.395	< 0.001
average monthly income (RMB)	4.918	0.725	0.244	6.784	< 0.001
Total score for adversity quotient	0.517	0.055	0.358	9.416	< 0.001
Total score for grit	0.075	0.014	0.203	5.559	< 0.001

*R*^2^ = 0.767, Adjusted *R*^2^ = 0.762, *F* = 147.927, *P* < 0.001.

## 5 Discussion

### 5.1 Analysis of the current status of nursing professional pride in blood purification centers and its variation across demographic characteristics

The results of this study show that the average score for nursing professional pride among hemodialysis nurses is (69.53 ± 15.21), which is at a moderate level. This is similar to the findings of Shim (Shim and Park, 2023). One possible reason is that poor mental health is associated with low nursing professional pride (Bildt and Michélsen, 2002). Prolonged clinical nursing work leads to a certain level of job stress and occupational burnout, resulting in reduced competence, lack of initiative, and a decrease in nursing professional pride (Acea-López et al., 2021). With the rapid development and widespread application of hemodialysis technology, the number of patients receiving hemodialysis treatment is increasing. Hemodialysis has become one of the most crucial means to sustain the lives of maintenance hemodialysis patients. However, the working environment for hemodialysis nurses is enclosed and highly specialized, and they frequently encounter special medical situations such as emergency dialysis and continuous bedside blood filtration. This leads to even more pronounced work-related stress (Lang et al., 2019). Ling et al. (2020) have found that hemodialysis nurses commonly experience occupational burnout, which can lead to a decrease in nursing professional pride. Nursing professional pride determines their motivation and willingness to provide care (Weis and Schank, 1997), and it is closely related to maintaining the quality of patient care, which has a positive impact on nurses' job satisfaction (Johnson et al., 2012). Therefore, how to enhance the level of nursing professional pride and job satisfaction, and improve the quality of nursing services, is a pressing issue that hospital authorities and nursing managers need to consider. In this study, the self-achievement dimension scored the lowest, which is in line with the findings of Silva-Junior et al. (2022). This may be attributed to the continuous high-paced work environment, leading hemodialysis nurses to develop a sense of meaninglessness in their work. Consequently, their work becomes mechanized and less efficient, resulting in decreased self-efficacy and reduced professional achievement. Nursing managers should assist nurses in establishing the correct professional values, implement hierarchical management to align their professional capabilities with their roles, and help them realize their sense of value. Additionally, enhancing communication skills among nursing staff can boost nurses' sense of achievement and increase their nursing professional pride (De Los Santos and Labrague, 2021; Liu et al., 2017).

Furthermore, the results of the multiple stepwise linear regression analysis indicate that education background, professional title, average monthly income are factors influencing nursing professional pride. In other words, the higher the education level, the higher the average monthly income, and the higher the professional title of hemodialysis nurses, the greater their nursing professional pride. Nurses with higher levels of education tend to have better self-regulation abilities and a stronger sense of professional identity (Wu et al., 2023). They are better able to identify their own emotions, the emotions of patients, and those of others around them, and they can enhance their work efficiency through effective emotional regulation. Additionally, nurses with higher levels of education tend to set higher standards for themselves and are more capable of achieving personal fulfillment through their clinical work (Li et al., 2021). These reasons are likely to contribute to an increase in nursing professional pride. It is recommended that managers pay more attention to employee growth, provide additional learning opportunities, promote continuing education, encourage nurses to pursue higher education, and enhance their specialized skills. Additionally, efforts should be made to help nursing graduates fully realize their value, fostering mutual development between the hospital and its staff (Berthelsen et al., 2019). There are reports suggesting that nurses with higher salaries and higher professional titles tend to have higher job satisfaction (Zhou et al., 2018). The satisfaction of nursing staff with their compensation and benefits can also impact their enthusiasm for work (Gerli et al., 2015). This may be a reason why nurses with higher professional title tend to have higher nursing professional pride. Furthermore, healthcare professionals with higher professional titles tend to be more satisfied with their work, possibly because nurses with higher professional titles may receive corresponding allowances and greater autonomy. This suggests that nursing managers should advocate for various benefits for nurses while also establishing a fair, just, and reasonable compensation distribution system to enhance their job satisfaction and motivation, ultimately leading to increased nursing professional pride and improved quality of nursing care (Zerden et al., 2022).

### 5.2 Correlation analysis of adversity quotient and nursing professional pride in blood purification centers

In this study, the average total adversity quotient score for hemodialysis nurses was (132.90 ± 41.10), indicating a moderate level. This is consistent with the findings of Wang et al. (2021). Due to the fact that the majority of respondents in this survey were females, their psychological and physical qualities tend to be inferior to those of males. When faced with emergency situations, females are more prone to nervousness, cognitive confusion, increased psychological stress, and lower levels of adversity quotient. In contrast, males often exhibit greater calmness, decisiveness, and quick thinking in psychological terms. Their response capabilities are typically more sensitive, allowing them to handle situations more effectively (Yu, 2021). At the same time, nurses are dealing with matters of life and have to bear a greater psychological burden. Societal disapproval and conflicts between nurses and patients can lead nurses to remain in a prolonged state of negativity, making it difficult for them to regain their composure. They may also tend to attribute the causes of adversity to themselves, feeling a limited sense of control over the reasons behind these adversities (Yan et al., 2023). Hemodialysis nurses are exposed to a long-term high-risk, high-demand work environment, and patients undergoing blood dialysis commonly experience anxiety and suffer from chronic illnesses. Nurses are consistently compelled to endure the emotional stress and intense stimuli brought on by patients (Hayes et al., 2015). Consequently, the prolonged fatigue from their work can lead nurses to react strongly to setbacks, struggle to attribute adversity to specific events, and find it challenging to remain composed in the face of adversity, resulting in significant negative consequences (Yu, 2021). Furthermore, in some hospitals, nursing management mechanisms are not fully developed, and nursing managers may overlook training related to adversity quotient, resulting in lower levels of adversity quotient among hemodialysis nurses.

The Pearson correlation analysis results indicated significant positive correlations between nurses' professional pride and all dimensions of adversity quotient, suggesting that higher adversity quotient levels in hemodialysis nurses are associated with stronger professional pride. Additionally, the multiple linear regression analysis results showed that adversity quotient is an influencing factor of nurses' professional pride. This suggests that the higher the level of adversity quotient among hemodialysis nurses, the stronger nursing professional pride. The adversity quotient theory holds that individuals exhibit varying responses when facing adversity or setbacks. The ability to accurately identify the causes of a situation, effectively address obstacles, and promptly learn from the experience when encountering adversity or challenges is referred to as adversity quotient (Stoltz and Wiley, 1997). It can guide individuals to summarize, condense, and synthesize a series of factors within the four stages of adversity occurrence, adversity recognition, adversity coping, and adversity impact. This allows for the quantification and systematization of an individual's resilience, helping them continuously recognize their shortcomings and improve their level of adversity quotient (Suryadi and Santoso, 2017). When hemodialysis nurses have a higher level of adversity quotient, they exhibit a greater sense of control over external factors and view adversity as an opportunity. They leverage their current adversity and their own efforts to gradually enhance their abilities, leading to increased confidence in problem-solving at work and a stronger sense of professional identity. Ultimately, this leads to a gradual increase in nursing professional pride (Li et al., 2022). Additionally, a higher level of adversity quotient enhances nurses' sense of responsibility, enabling them to take effective measures to turn situations around in the face of setbacks. They also receive recognition and acknowledgment from others in their work, further enhancing nursing professional pride (Wang et al., 2021).

The level of adversity quotient in nurses is not only related to their personal physical and mental health but also determines their ability to perform nursing work competently. Improving adversity quotient not only enhances nurses' ability to respond to adversity but also increases their enthusiasm for work, ultimately strengthening organizational competitiveness (Yu et al., 2021). Research has found that adversity quotient can boost nurses' self-efficacy, reduce feelings of professional burnout, and thereby enhance nursing professional pride (Li et al., 2022). Furthermore, there is a correlation between adversity quotient and nurses' work engagement. Researchers like Brubakk et al. (2019) have found that low levels of work engagement can lead to reduced nursing work efficiency, ultimately resulting in decreased job satisfaction, decreased nursing professional pride, and an impact on the quality of nursing care. Therefore, managers can enhance nursing professional pride from the perspective of adversity quotient. First, hospital nursing managers should take into full consideration the personality traits, personal values, and needs of each nurse when organizing work, allowing individuals to realize their potential (Liang et al., 2016). They can develop various forms of educational and training programs related to adversity quotient, including theoretical knowledge training on adversity, sharing of adversity scenarios and coping methods, simulation training and assessment of adversity scenarios (Huang et al., 2021). These measures can strengthen nurses' psychological coping abilities in facing adversity, overcoming it, and transcending it; Furthermore, universities can establish a comprehensive adversity quotient assessment system by increasing the inclusion of courses related to humanities and character development. They can introduce teaching methods such as scenario-based learning, group activities, and flipped classrooms to proactively nurture nurses' ability to handle adversity. This early training can facilitate a smooth transition for new nurses as they enter their roles (Wei et al., 2022). This approach aims to enhance nurses' adversity quotient, boost their nursing professional pride, and improve their ability to cope with stress.

### 5.3 Correlation analysis of grit and nursing professional pride in blood purification centers

In this study, the average total grit score for hemodialysis nurses was (30.95 ± 10.54), indicating a moderate level. This is consistent with the findings of researchers such as Shin (2022) and Fernández-Miranda et al. (2023). Clinical nursing is an incredibly challenging and high-risk profession. Nurses not only have to directly confront patients' illnesses or even death but also have to deal with the various forms of suffering experienced by patients, their families, and caregivers in their daily work (Radfar et al., 2021). Moreover, the sharp increase in natural and man-made disasters has brought about changes and challenges to healthcare, imposing additional social and psychological burdens on nurse (Deng et al., 2019). Several studies have indicated that high levels of stress, such as anxiety and depression, exist among healthcare workers worldwide (Alhroub et al., 2022; Chen et al., 2022; Gül and Kiliç, 2021). Furthermore, research has shown that occupational psychological distress, such as anxiety, is negatively correlated with grit levels (Peng and Wu, 2022). The highly demanding and emotionally charged nature of the work performed by hemodialysis nurses can further increase their physical and emotional stress, leading to psychological distress such as anxiety and depression, which in turn can result in decreased grit levels.

The Pearson correlation analysis results revealed significant positive correlations between nurses' professional pride and all dimensions of resilience, indicating that higher resilience levels in hemodialysis nurses are associated with stronger professional pride. Furthermore, the multiple linear regression analysis results indicated that resilience is an influencing factor of nurses' professional pride. This suggests that the higher the level of grit among hemodialysis nurses, the stronger nursing professional pride. Positive psychology theory advocates starting with an individual's inherent potential positive strengths and courage. It encourages viewing the essence of things with a positive mindset to tap into people's innate or actual positive qualities and strengths. This approach helps individuals move toward the shores of happiness (Seligman, 2000). Research on this theory primarily focuses on three aspects: positive emotions and experiences, positive personality traits, and a positive social environment (Proyer et al., 2014). Grit, as a positive personality trait, can provide positive psychological support to hemodialysis nurses. It allows them to view issues from a positive perspective, enhance their problem-solving abilities, reduce personal negative emotional experiences, and strengthen their professional pride (Datu, 2021). It manifests as a commitment to work and maintaining sustained interest, playing a positive role in subjective wellbeing and psychological health. Furthermore, nurses' grit can influence their job satisfaction. Individuals with high levels of grit consistently maintain interest in their goals and continue to work diligently. They also adapt their behavior as needed to achieve those goals (Cho and Kim, 2022). Therefore, grit can enhance the job satisfaction of hemodialysis nurses and increase their nursing professional pride.

Grit is the driving force behind overcoming failures, adversity, and setbacks to achieve long-term goals. It is a crucial component of achieving objectives and talents. It enables nurses to progress toward long-term goals even in challenging situations without giving up (Wang, 2021). Grit is a predictor of success and psychological wellbeing (Jeong et al., 2019). Nurses with higher levels of grit have stronger clinical abilities and lower levels of occupational burnout (Seguin, 2019; Ko and Gu, 2020). Furthermore, Cho and Kim (2022) found that nurses with higher levels of grit also have higher job satisfaction and provide higher-quality clinical care. Therefore, managers can enhance nursing professional pride from the perspective of grit. Healthcare institutions should coordinate nursing tasks, establish reasonable career management systems, and implement resilience enhancement programs to improve nurses' psychological resources and work dedication, allowing them to be fully engaged in their work (Jeong et al., 2019). Related research has reported correlations between grit and occupational psychological distress, hope levels, and social support (Yang and Wu, 2021). Therefore, managers can implement intervention measures, such as organizing lectures on positive psychology, conducting timely psychological counseling and crisis interventions to reduce negative emotions among hemodialysis nurses, enhance their levels of hope, and strengthen their social support. This, in turn, can lead to an increase in grit levels and boost nursing professional pride.

### 5.4 Strengths and limitations

This study has several advantages as follows: Firstly, it employs the nursing professional pride scale to investigate nursing professional pride. Secondly, it reports the correlation between nursing professional pride, adversity quotient, and grit, a field with relatively limited research. Thirdly, the research findings of this study can serve as a theoretical basis for scholars to develop interventions aimed at enhancing nursing professional pride and increasing their sense of professional identity.

However, this study still has some limitations: Firstly, this study focused solely on nurses in blood purification centers in Sichuan Province; But it can provide some reference for nurses in similar high-pressure environment. Future studies could still expand the sample size to include nurses from different provinces and departments, investigating other potential influencing factors of professional pride to see if these findings apply to other nursing specialties or work environments. Secondly, while this study explored the influencing factors of nurses' professional pride, it did not implement any intervention measures. Future research could design and implement targeted interventions to enhance professional pride and improve nursing quality. Lastly, although this study included several key variables in analyzing influencing factors, there is still room for expansion. Future studies should aim to construct a more comprehensive analytical framework that includes additional dimensions, such as work environment, social support, and organizational culture, for a thorough analysis of the influencing factors on hemodialysis nurses' professional pride.

## 6 Conclusion

This study indicates that nurses in blood dialysis centers exhibit moderate levels of nursing professional pride, adversity quotient, and grit. Education background, professional title, average monthly income adversity quotient, and grit were identified as factors influencing nursing professional pride. The study confirms the correlation between nursing professional pride and adversity quotient, and grit. In the future, administrators can implement interventions from the perspective of adversity quotient and grit to enhance their nursing professional pride.

## Data Availability

The raw data supporting the conclusions of this article will be made available by the authors, without undue reservation.
